# Risk of Type 2 Diabetes in University Students at the University of Extremadura: A Cross-Sectional Study

**DOI:** 10.3390/jpm14020146

**Published:** 2024-01-29

**Authors:** Pilar Alfageme-García, Belinda Basilio-Fernández, María del Valle Ramírez-Durán, Adela Gómez-Luque, Víctor Manuel Jiménez-Cano, Juan Fabregat-Fernández, Vicente Robles Alonso, María Zoraida Clavijo-Chamorro, Sonia Hidalgo-Ruíz

**Affiliations:** 1 Department of Nursing, University Center of Plasencia, University of Extremadura, 10600 Plasencia, Spain; palfagemeg@unex.es (P.A.-G.); valleramirez@unex.es (M.d.V.R.-D.); adelagl@unex.es (A.G.-L.); victormajc@unex.es (V.M.J.-C.); juanfabregat@unex.es (J.F.-F.); vroblesa@unex.es (V.R.A.); kirosony@unex.es (S.H.-R.); 2 Department of Nursing, Faculty of Nursing and Occupational Therapy, University of Extremadura, 10004 Cáceres, Spain; zoraidacc@unex.es

**Keywords:** diabetes risk, university students, Findrisk test, modifiable risk factors, diabetes mellitus type 2

## Abstract

The prevalence of type 2 diabetes is increasing worldwide. The aim of our study was to detect people susceptible to DM among a university population aged 18 to 45 years and analyze the existence of modifiable risk factors in order to implement prevention programs, in addition to analyzing BMI data related to the variables under study. We proposed a descriptive, cross-sectional study following the recommendations of cross-sectional studies (STROBE), with a sample of 341 subjects, students enrolled at the University of Extremadura, carried out by two researchers. The research protocol was approved by the Bioethics Committee of the University of Extremadura (165/2021). The study considered the Findrisk questionnaire in Spanish, validated by the Blackboard Study, a stadiometer to measure height, a bioimpedance meter to evaluate weight and body composition parameters, and a blood pressure monitor to measure blood pressure. The results indicated that the participants had a low risk of suffering T2DM. The highest Findrisk test scores were found in those with a BMI value above 25, lower physical activity, poor dietary intake of fruits and vegetables, and increased fat mass. Our future research will be the implementation of T2DM prevention programs, acting on modifiable factors.

## 1. Introduction

Diabetes mellitus (DM) is a metabolic disorder characterized by chronic hyperglycemia and disorders in the metabolism of carbohydrates, fats, and proteins due to defects in the secretion and/or action of insulin [1]. It has a high prevalence and a strong tendency to develop both acute and chronic complications that decrease the quality of life and life expectancy of those who suffer from it, leading to a high economic, social and health impact [2].

The International Diabetes Federation (IDF) estimates the global obesity prevalence in the population aged 20 to 79 years to be 10.5% worldwide, with rates of 9.2% in Europe, and 10.3% in Spain. This means that 536.6 million people have currently been diagnosed with DM worldwide, with 61.4 million in Europe and 5.1 million in Spain. The IDF forecasts for 2045 are not very favorable, with increases in the global prevalence to 12.2%, which will mean that more than 783 million people in the world will have DM by that date. These figures are even more alarming if we consider that undiagnosed DM is estimated to be as high as 44.7%, which would mean that almost one-in-two people are unaware of having DM. It is not surprising then, that due to its rapid growth it is considered a health emergency [3], having elevated to the category of epidemic in the 21st century [4,5] and representing one of the highest priority health problems [2].

Of the existing types of diabetes mellitus, Type 2 DM (T2DM) is the most prevalent, representing approximately 90–95% of DM cases [6]. Although its causes have yet to be fully clarified, we know that it originates from a multigenic predisposition triggered by environmental and behavioral factors, with a close relationship with overweight, obesity, increasing age, alterations in glucose regulation, certain ethnic groups, family history, unhealthy dietary patterns, and a sedentary lifestyle [3,7].

Several studies have confirmed the efficacy of early diagnosis and intensive lifestyle interventions in people at high risk of developing T2DM [8,9,10,11]. Early detection becomes even more important when scientific evidence demonstrates the existence of micro- and/or macrovascular complications at the diagnosis of T2DM [12] that could stop or slow its progression if detected early [8]. However, despite the increase in the incidence of T2DM in the younger population [13,14,15,16], it is often omitted from screening. Agencies such as the American Diabetes Association (ADA) only advise screening in those under 45 years when there is overweight or obesity and some risk factor (a maternal history of gestational diabetes, family history, race/ethnicity, and/or signs of insulin resistance) [6].

The use of self-administered clinical prediction rules is established as a simple and inexpensive tool that can facilitate screening in individuals who are not usually targeted in the screening process [17]. In addition, it can select those individuals susceptible to apply blood tests and subsequent educational and reinforcement interventions in the prevention of T2DM, making screening simpler and more efficient [18], which will enable reductions in its incidence [9,18,19]. In this regard, the Findrisk test is widespread worldwide and has been validated in numerous countries as an efficient tool with high diagnostic performance for the detection of diabetes at 10 years [20,21]. In Spain, the Pizarra study validated the test in a population of southwestern Spain, demonstrating a positive predictive value of 22.2% and a negative predictive value of 96% [17]. For its part, the DE-PLAN study, developed in Catalonia, demonstrated a sensitivity of 75.9% and 65.8% for T2DM and prediabetes, respectively, and a specificity of 52.3% for T2DM and 56.7% for T2DM and 56.7% for prediabetes, respectively. The negative predictive value for T2DM was 95.5% and 78.4% for prediabetes [9,22].

The aim of our study was to carry out a descriptive study of the application of the Findrisk test in a population of university students aged 18 to 45 years in Extremadura (Spain) to detect people susceptible to T2DM 10 years ahead and to detect whether there are risk factors which can influence its prevention, in addition to analyzing the BMI related to the different variables under study.

Our study is novel in that it focuses on university students aged 18 to 45 years, whereas similar studies in the literature that have been carried out in university populations have been with academics and employees, not only including students [23].

## 2. Materials and Methods

### 2.1. Research Design

A descriptive, cross-sectional observational study was conducted following the recommendations for strengthening reports of observational studies in epidemiology (STROBE) [24]. The Findrisk screening of the participants who met the inclusion criteria was performed by 3 nurses from the research group (GICISA), nurses of the Nursing Department, University Center of Plasencia, University of Extremadura, following a protocol designed to avoid inter-professional bias.

### 2.2. Participants

**Figure 1:**
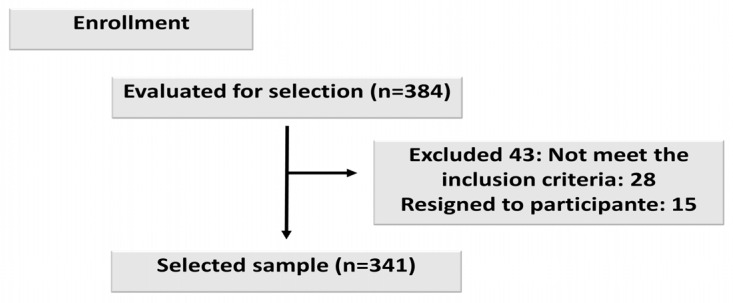
Participants.

In total, 384 people participated in the study. Of those, 43 were excluded due to missing data. The final sample consisted of a total of 341 subjects (Figure 1). Recruitment was carried out at the University of Extremadura, from November 2022 to March 2023.

The inclusion criteria for participation in the research were those 18–45 years of age, enrolled at the University of Extremadura. Exclusion criteria were a previous diagnosis of diabetes, mobility disability and/or comorbidities incompatible with moderate exercise, and not being a student.

The participants were contacted through a message to their institutional e-mail where they received an explanation of the project and a link to the informed consent form where they could accept their participation. Another means of recruitment was the placement of informative posters in the university centers, with a QR code linking to information explaining the project and informed consent, as well as the places, days of measurement, and data collection, to which participants signed up through a link. The research staff contacted the participants by telephone a few days before data collection. In addition, the research group reinforced the information about the project with visits during class hours and dissemination on social networks (Facebook and Instagram).

The sample size was calculated according to Laid and Kelley [25]; a minimum of 340 subjects would be needed to develop the study with a representative sample, taking into account the total population enrolled in the campus of Plasencia and Cáceres, with a confidence level of 95%.

The research protocol was approved by the Bioethics Committee of the University of Extremadura (165//2021), in accordance with the Declaration of Helsinki and national legislation on bioethics, biomedical research, and sample confidentiality.

### 2.3. Measuring Instrument and Procedure

The Spanish version of Findrisk, a Finnish questionnaire that includes T2DM risk factors and estimates the risk of suffering T2DM at 10 years’ time, was used for the study. This questionnaire has been validated by the Pizarra Study in a Spanish population aged between 18 and 65 years. The results showed a positive predictive value of 22.2% and a negative predictive value of 96% [18]. The DE-PLAN study, also developed in Spain, showed a sensitivity of 75.9% and a diagnostic specificity of T2DM of 52.3% and a diagnostic specificity of prediabetes of 65.8% [9,22]. Furthermore, its usefulness has been demonstrated in numerous international studies [20,21,26,27,28].

Findrisk screening of the participants who met the inclusion criteria was performed by 3 nurses from the research group, using a protocol designed to avoid interprofessional bias. The test included 8 items: age, body mass index (weight and height), waist circumference, daily physical activity, frequency of eating vegetables and fruits, hypertension, a history of hyperglycemia, and a family history of diabetes.

A stadiometer (Seca 213) was used to measure height; a Tanita Body Composition Analyzer bioimpedance meter (Tanita MC 780 MA) was used to evaluate weight and the body composition parameters used in the study; and a brachial blood pressure monitor (microlife watch bp office) was used to measure BP in both arms simultaneously.

### 2.4. Statistical Analisis

Descriptive statistics were calculated. All statistical analyses were performed using IBM SPSS statistics version 27.0.

Personal information obtained in this study was anonymized by coding; no information identifying participants has been stored. Only the investigators had access to the final trial dataset. All data from this study will be securely managed for 5 years after the completion of the study. Subsequently, the materials will be deleted.

Participants could withdraw consent to participate at any time. There was no disenrollment. There were no reasons for excluding any participant, except those mentioned in the exclusion criteria, which has minimized the bias affecting the study results.

## 3. Results

In total, 341 subjects (209 females (61.3%) and 97 males (38%), mean age 23.54 ± 5.92) (between 18 and 45 years) were included in this study. The study participants exhibited a mean weight of 64.49 ± 13.06 and a mean height of 166.25 ± 9.31. The BMI presented by our participants was 23 ± 4.52. The Findrisk score is presented as a mean of 4.39 ± 3.25. Over half (51.9%) of the sample performed less than 30 min of physical activity; 57.8% reported eating fruit and vegetables every day.

The risk of diabetes in our participants was low in 78.9% of the cases compared with 0.6% of participants who were at high risk, while 16.1% presented a slightly low risk and 4.4% presented a moderate risk. This means that 4.98% were at moderate or high risk of developing diabetes in the next 10 years Table 1.

All the characteristics of the study are shown in Table 2.

In the present study, there were significant differences between women and men with respect to the risk of suffering from diabetes, with women presenting a higher value than men. This may be due to the fact that in our study, men reported more physical activity than women. There were also significant differences between those who ate fruit or vegetables every day and those who did not, as well as by age and BMI. Those under 25 years of age showed significant differences from those over 25 years of age, having a lower risk of diabetes, as did the participants with a higher BMI, whose score was significantly higher compared with those with a lower BMI, as shown in Table 3. 

The BMI presented an average of 23.40 ± 4.53 (kg/m^2^), with the BMI in men being 24.19 ± 3.48 (Kg/m^2^) and in women 23.03 ± 4.90 (kg/m^2^) As shown in the following table, participants older than 25 years had a higher BMI and the difference in BMI by sex was significantly lower in women.

The relationship between BMI and the variables measured is presented in Table 4.

Analyzing the results by sex (Table 5), it appears that women have higher average values of fat mass (28.56) compared with men, who have an average of 20.27. On the other hand, women have lower values compared with men in the percentage of fat-free mass, and in the percentage of lean mass.

In the correlation analysis (Supplementary Materials), a positive linear correlation was found between FINDRISK scores and the percentage of fat mass, and negative linear correlations were found between FINDRISK scores and metabolic rate, the percentage of fat-free mass, the percentage of lean mass, the percentage of body water and also with the percentage of bone mineral. The correlation coefficient, r, was positive, i.e., 0 < r <1; furthermore, the closer the coefficient is to 1, the stronger the correlation, and the closer it is to 0, the less correlation. If the correlation coefficient, r, is negative, the correlation is negative or inverse (when one variable increases, the other decreases), i.e., −1 < r < 0; furthermore, the closer it is to −1, the stronger the correlation is, and the closer it is to 0, the less correlation there is between the variables.

In our study, the relationship between overweight and diabetes was maintained, as was the case with BMI > 25 kg/m^2^: the higher the BMI, the greater the risk of suffering from diabetes.

In the correlation analysis, a moderate linear correlation was found between FINDRISK scores and age.

## 4. Discussion

This study is the first to be carried out in our setting, with university students, using the Findrisk test for the 10-year prediction of T2DM, in a sample aged between 18 and 45 years old. The main reason for initiating screening in this population is the increase in the incidence of T2DM in the younger population [13,14,15,16]. We know that organizations such as the ADA advise screening in people under 45 years of age when there is a presentation of overweight or obesity and some risk factor [6]. In our study, we detected 17 individuals at moderate–high risk of T2DM; 4.98% of the sample with risk factors related to weight gain and physical inactivity, which leads us to consider educational interventions to act on these modifiable risk factors.

The obtained Findrisk scores are presented as means of 4.39 ± 3.25. In this sense, most of the studies we have found on the risk of developing diabetes obtained higher scores, varying between the 7 points obtained by Yildiz et al. [23] and the 12.42 attained by Cevik et al. [29]. This discrepancy is due to the population studied and the mean age of our sample. What we did find in common with the rest of the literature is that the risk of developing diabetes increases with age, physical inactivity, and the presence of overweight and obesity [23,29,30,31].

In our study, the possibilities of suffering from T2DM prevailed in women; in this sense, there was variability in findings in other studies; in their studies, Yildiz et al. and Cevik et al. describe that men are more at risk [23,29], but there are others, such as that of Ephraim et al. [30], which found that there are more women with prediabetes and diabetes than men. We can justify these data with two variables of the study: the first is that in the sample studied, men perform more physical activity; the second is that women have a higher percentage of fat mass than men. In turn, we can affirm that in the sample studied, the risk of developing T2DM is related to the increase in body fat mass and the decrease in bone mineral or metabolic rate; to date, we have not found any study in the scientific literature that relates these variables to the risk of suffering from T2DM.

In our research, there was a clear relationship between an increased BMI and the risk of T2DM; it is a known and studied fact that people with a BMI ≥25 kg/m^2^ have an increased risk of DM, as it appeared in the 2023 diabetes standards of care [6]. Overweight and obesity, two of the most important risk factors in the development of T2, is a public health problem, and its prevalence is steadily increasing worldwide [32]. According to data collected in studies on the effect of overweight and obesity in 195 countries over 25 years, the frequency of obesity is higher in women [33]; in the study by Sonmez et al., they agree that the frequency of obesity is higher in women [34]. However, the result in our study differs since the percentage of women with overweight and obesity was 22.7% (*n* = 71), and that of men was 34.4% (*n* = 117).

Among the risk factors that make up the Findrisk test are health behaviors such as physical activity and nutrition, specifically the consumption of fruits and vegetables. These modifiable factors will directly influence other risks assessed in the test that can also be improved, such as weight and waist circumference. In addition, they will positively influence glucose and hypertension levels [33,34]. Despite the possible improvement in both values, if, at the time of screening, the patient has already presented some hyperglycemia or has taken hypertensives, their score would not decrease in a future Findrisk test; however, their health would benefit as both physical exercise and a healthy diet would help them to maintain better levels.

The importance of physical activity for the prevention of T2DM has been recognized in many studies [35,36,37,38,39,40,41]. The ADA 2024 recommendations for T2DM prevention are performing moderate physical activity of more than 150 min/week, such as brisk walking, and the inclusion of resistance exercises [33]. In our study, despite our population being young, it was surprising to see that 51.9% (*n* = 176) of the sample performed less than 30 min of physical activity per day. Our results are similar to those of Kulak et al.; in their study, physical inactivity accounted for 59% of the sample [42]. However, our results differ from other work conducted among academics and university workers where only 13.3% of the participants were not physically active; our population was younger but much less physically active. In our university, there is a physical activity and sports service (Safyde) in charge of promoting and organizing those activities aimed at satisfying the physical activity and sports needs of the members of the university community; however, the results of our study highlight the need to continue working to prevent physical inactivity among our students.

In terms of diet, fruit and vegetable consumption is associated with a lower risk of developing T2DM [43,44,45,46]. In our population, 57.8% (*n* = 198) reported that they consumed fruits and vegetables every day, and 42.2% (*n* = 143) did not. These results are similar to those found in studies conducted in other age groups, such as that of Kulak et al. or Yildiz et al., in which 39.8% and 46.8% did not consume fruits and vegetables on a regular basis, respectively [23,42]. It is important to work on activities to promote healthy eating habits which, as the scientific evidence indicates, focus on the overall quality of the food consumed and which, in addition to the consumption of fruits and vegetables, include other foods such as whole grains, legumes and nuts [43,44,45,46]. Refined and processed foods should be reduced to a minimum, controlling weight, if necessary, with a macronutrient distribution based on individualized assessments of dietary patterns, preferences, and metabolic objectives that can prevent T2DM [33,47].

The practical applications of this study include understanding the disease, understanding its risk factors, highlighting the modifiable risk factors, and being able to carry out T2DM prevention programs, this being the next line of work.

The main limitation is that this study was cross-sectional, and we could not analyze the cause–effect factor. The sample size was further limited by the number of students at the University of Extremadura. A small sample of students was assessed; in future research, we plan to increase the sample.

## 5. Conclusions

The highest Findrisk test scores were found in those with a BMI above 25, which translates into overweight individuals, as well as those with lower physical activity levels, poor dietary intakes of fruits and vegetables, and increased fat mass. These risk factors were present in university students in Extremadura that could increase the risk of T2DM. Our future research will be aimed at implementing T2DM prevention programs, acting on modifiable factors.

## Figures and Tables

**Table 1:** Diabetes risk results according to Findrisk score limits.

TOTAL SCORE	INTERPRETATION	RESULTS OF OUR RESEARCH
Less than 7 points	Low risk level	78.9%
From 7 to 11 points	Slightly low risk level	16.1%
From 12 to 14 points	Moderate risk level	4.4%
From 15 to 20 points	High risk level	0.6%
More than 20 points	Very high risk level	0%

**Table 2:** Demographical characteristics of the study participants.

QUANTITATIVE VARIABLES	
	Mean ± SD
Age (years)	23.54 ± 5.93
Height (cm)	166.26 ± 9.32
Basal Metabolism (kcal)	1478.46 ± 275.02
Weight (kg)	64.50 ± 13.06
% Fat Mass	25.95 ± 7.40
% Fat-Free Mass	73.98 ± 7.47
% Lean Mass	69.48 ± 9.91
Body Water %	53.25 ± 5.76
Body Mineral %	3.72 ± 0.53
BMI (kg/m^2^)	23.40 ± 4.53
Sarcopenia Index Risk	7.03 ± 1.21
Visceral Fat Index	3.08 ± 2.87
Metabolic Rate	7.66 ± 2.22
Waist Circumference (cm)	76.83 ± 13.25
Hip Circumference (cm)	101.09 ± 9.55
Waist/Hip Ratio	0.76 ± 0.09
Findrisk Score	4.39 ± 3.25
	
QUALITATIVE VARIABLES	n (%)
SEX	
Female	209 (68.3%)
Male	97 (31.7%)
PHYSICAL ACTIVITY	
<30 min	177 (51.9%)
>30 min	164 (48.1%)
FRUIT AND VEGETABLES	
No todos los días	144 (42.2%)
Todos los días	197 (57.8%)
HYPERTENSION MEDICATION	
NO	335 (98.2%)
YES	6 (1.8%)
GLUCOSE	
NO	331 (97.1%)
YES	10 (2.9%)
FAMILY HISTORY OF DIABETES	
No	171 (50.3%)
Parents, siblings, children, etc.	36 (10.6%)
Grandparents, aunts, uncle, cousins, etc.	131 (38.5%)
Both (grandparents, parents, etc.)	2 (0.6%)
RISK	
Low	269 (78.9%)
Slightly Elevated	55 (16.1%)
Moderate	15 (4.4%)
High	2 (0.6%)
Very High	(0%)

**Table 3:** Findrisk score of the participants according to the variables measured.

		SCORE		Statistical Test
		Average	Deviation	*p*-Value
SEX	Female	4.61	3.12	<0.01
	Male	3.70	3.22	
PHYSICAL ACTIVITY	<30 min	5.44	2.98	<0.01
	>30 min	3.26	3.14	
FRUIT AND VEGETABLES	Not every day	5.16	3.01	<0.01
	Every day	3.83	3.31	
HYPERTENSION MEDICATION	No	4.33	3.22	>0.05
	Yes	8.00	3.16	
GLUCOSE	No	4.24	3.13	>0.05
	Yes	9.50	3.10	
FAMILY HISTORY OF DIABETES	No	2.37	2.22	>0.05
	Parents, siblings, children	8.25	2.56	
	grandparents, aunts, uncle, cousins	5.88	2.69	
	Both (grandparents, parents)	9.50	2.12	
AGE (years)	<25	4.01	2.99	<0.01
	>25	6.00	3.26	
BMI (kg/m^2^)	Thin	3.00	2.37	<0.01
	Normal weight	3.56	2.58	
	Overweight	5.72	3.31	
	Obese	10.33	2.58	

**Table 4:** BMI results according to the variables under study.

		BMI			
		Thin	Normal Weight	Overweight	Obese
		% of the Row *n*	% of the Row *n*	% of the Row *n*	% of the Row *n*
SEX	Female	9.7%	67.6%	17.4%	5.3%
	Male	1.0%	64.6%	30.2%	4.2%
PHYSICAL ACTIVITY	<30 min	7.7%	68.6%	18.6%	5.1%
	>30 min	6.1%	64.6%	24.5%	4.8%
FRUIT VEGETABLES	Not every day	5.4%	68.5%	22.3%	3.8%
	Every day	8.1%	65.3%	20.8%	5.8%
HYPERTENSION MEDICATION	NO	7.0%	66.3%	21.7%	5.0%
	YES	0.0%	100.0%	0.0%	0.0%
GLUCOSE	NO	6.8%	66.7%	21.4%	5.1%
	YES	11.1%	66.7%	22.2%	0.0%
FAMILY HISTORY OF DIABETES	NO	9.7%	64.3%	22.1%	3.9%
	Parents, siblings, children	0.0%	62.1%	24.1%	13.8%
	Grandparents, aunts, uncles, cousins	4.3%	70.9%	20.5%	4.3%
	Both (grandparents, parents)	50.0%	50.0%	0.0%	0.0%
AGE (years)	<25	10.5%	69.8%	16.7%	3.1%
	>25	0.0%	57.1%	30.4%	12.5%

**Table 5:** Analysis of body parameters by sex.

	SEX			
	Female		Male	
	Average	Deviation	Average	Deviation
% Fat mass	28.56	6.34	20.27	6.30
% Fat-free mass	71.37	6.43	79.69	6.33
% Lean mass	67.38	7.66	74.03	12.43
% Body water	51.32	5.17	57.41	4.68
% Bone mineral	3.63	0.44	3.91	0.65
BMI (kg/m^2^)	23.03	4.90	24.19	3.48

## Data Availability

The raw data supporting the conclusions of this article will be made available by the authors on request.

## Supplementary Materials

The following supporting information can be downloaded at: https://www.mdpi.com/article/10.3390/jpm14020146/s1, Correlation tables and crosstab.

## References

[1] World Health Organization (1999). Definition, Diagnosis and Classification of Diabetes Mellitus and Its Complications: Report of a WHO Consultation. Part 1: Diagnosis and Classification of Diabetes Mellitus.

[2] (2020–2024). Extremadura Govern: Integral Plan in Extremadura. https://www.fadex.org/bddocumentos/QBDTB-PIDIA-2020-2024.pdf.

[3] International Diabetes Federation IDF Diabetes Atlas 2021.

[4] Formiga F., Camafort M., Carrasco-Sánchez F.J. (2020). Heart failure and diabetes: The confrontation of two major epidemics of the 21st century. Rev. Clin. Esp..

[5] Standl E., Khunti K., Hansen T.B., Schnell O. (2019). The global epidemics of diabetes in the 21st century: Current situation and perspectives. Eur. J. Prev. Cardiol..

[6] American Diabetes Association (2023). Standards of Medical Care in Diabetes 2023. Diabetes Care.

[7] Ezkurra Loiola P. (2016). Fundación Red GDPS. Guía de Actualización en Diabetes Mellitus Tipo 2.

[8] Vermunt P.W.A., Milder I.E.J., Wielaard F., de Vries J.H.M., Baan C.A., van Oers J.A.M., Westert G.P. (2012). A lifestyle intervention to reduce type 2 diabetes risk in Dutch primary care: 2.5-year results of a randomized controlled trial. Diabet. Med..

[9] Costa B., Barrio F., Cabré J.J., Piñol J.L., Cos X., Solé C., Bolíbar B., Basora J., Castell C., Solà-Morales O. (2012). Delaying progression to type 2 diabetes among high-risk Spanish individuals is feasible in real-life primary healthcare settings using intensive lifestyle intervention. Diabetologia.

[10] Balk E.M., Earley A., Raman G., Avendano E.A., Pittas A.G., Remington P.L. (2015). Combined diet and physical activity promotion programs to prevent type 2 diabetes among persons at increased risk: A systematic review for the community preventive services task force. Ann. Intern. Med..

[11] Stevens J.W., Khunti K., Harvey R., Johnson M., Preston L., Woods H.B., Davies M., Goyder E. (2015). Preventing the progression to type 2 diabetes mellitus in adults at high risk: A systematic review and network meta-analysis of lifestyle, pharmacological and surgical interventions. Diabetes Res. Clin. Pract..

[12] UK Prospective Diabetes Study (UKPDS) (1999). VIII. Study design, progress and performance. Diabetologia.

[13] Rosenbloom A.L., Joe J.R., Young R.S., Winter W.E. (1999). Emerging epidemic of type 2 diabetes in youth. Diabetes Care.

[14] Pettitt D.J., Talton J., Dabelea D., Divers J., Imperatore G., Lawrence J.M., Liese A.D., Linder B., Mayer-Davis E.J., Pihoker C. (2014). Prevalence of diabetes in U.S. youth in 2009: The SEARCH for diabetes in youth study. Diabetes Care.

[15] Lawrence J.M., Imperatore G., Pettitt D.J., Dabelea D., Linder B., Mayer-Davis E.J., Isom S., Pihoker C., Standiford D.A., Marcovina S.M. (2014). Incidence of diabetes in United States youth by diabetes type, race/ethnicity, and age, 2008–2009. Diabetes.

[16] Bjornstad P., Drews K.L., Caprio S., Gubitosi-Klug R., Nathan D.M., Tesfaldet B., Tryggestad J., White N.H., Zeitler P. (2021). TODAY Study Group. Long-Term Complications in Youth-Onset Type 2 Diabetes. N. Engl. J. Med..

[17] Ramírez-Durán M.d.V., Basilio-Fernández B., Gómez-Luque A., Alfageme-García P., Clavijo-Chamorro M.Z., Jiménez-Cano V.M., Fabregat-Fernández J., Robles-Alonso V., Hidalgo-Ruiz S. (2022). Efficacy of an Online Educational Intervention in Reducing Body Weight in the Pre-Diabetic Population of 18–45 Years Old, a Randomized Trial Protocol. J. Pers. Med..

[18] Soriguer F., Valdés S., Tapia M.J., Esteva I., Ruiz De Adana M.S., Almaraz M.C., Morcillo S., Fuentes E.G., Rodríguez F., Rojo-Martinez G. (2012). Validación del FINDRISC (FINnish Diabetes Risk SCore) para la predicción del riesgo de diabetes tipo 2 en una población del sur de España. Estudio Pizarra. Med. Clin..

[19] García J. (2018). Guía de Diabetes Tipo 2 Para Clínicos. Recomendaciones de la redGDPS. Fundación redGDPS. https://www.redgdps.org/guia-de-diabetes-tipo-2-para-clinicos/introduccion-20180907.

[20] Franciosi M., De Berardis G., Rossi M.C., Sacco M., Belfiglio M., Pellegrini F., Tognoni G., Valentini M., Nicolucci A. (2005). Use of the diabetes risk score for opportunistic screening of undiagnosed diabetes and impaired glucose tolerance: The IGLOO (Impaired Glucose Tolerance and Long-Term Outcomes Observational) study. Diabetes Care.

[21] Makrilakis K., Liatis S., Grammatikou S., Perrea D., Stathi C., Tsiligros P., Katsilambros N. (2011). Validation of the Finnish diabetes risk score (FINDRISC) questionnaire for screening for undiagnosed type 2 diabetes, dysglycaemia and the metabolic syndrome in Greece. Diabetes Metab..

[22] Costa B., Barrio F., Piñol J.L., Cabré J.J., Mundet X., Sagarra R., Salas-Salvadó J., Solà-Morales O. (2013). DE-PLAN-CAT/PREDICE Research Group. Shifting from glucose diagnosis to the new HbA1c diagnosis reduces the capability of the Finnish Diabetes Risk Score (FINDRISC) to screen for glucose abnormalities within a real-life primary healthcare preventive strategy. BMC Med..

[23] Yildiz T., Zuhur S., Shafi Zuhur S. (2021). Diabetes Risk Assessment and Awareness in a University Academics and Employees. Med. Bull. Sisli Etfal Hosp..

[24] Vandenbroucke J.P., von-Elm E., Altman D.G., Gøtzsche P.C., Mulrow C.D., Pocock S.J., Poole C., Schlesselman J.J., Egger M. (2014). STROBE Initiative. Strengthening the Reporting of Observational Studies in Epidemiology (STROBE): Explanation and elaboration. Int. J. Surg..

[25] Lai K., Kelley K. (2012). Accuracy in parameter estimation for ANCOVA and ANOVA contrasts: Sample size planning via narrow confidence intervals. Br. J. Math. Stat. Psychol..

[26] Bergmann A., Li J., Wang L., Schulze J., Bornstein S.R., Schwarz P.E. (2007). A simplified Finnish diabetes risk score to predict type 2 diabetes risk and disease evolution in a German population. Horm. Metab. Res..

[27] Schwarz P.E., Lindström J., Kissimova-Scarbeck K., Szybinski Z., Barengo N.C., Peltonen M., Tuomilehto J. (2008). DE-PLAN project. The European perspective of type 2 diabetes prevention: Diabetes in Europe—Prevention using lifestyle, physical activity and nutritional intervention (DE-PLAN) project. Exp. Clin. Endocrinol. Diabetes.

[28] Schwarz P.E., Gruhl U., Bornstein S.R., Landgraf R., Hall M., Tuomilehto J. (2007). The European Perspective on Diabetes Prevention: Development and Implementation of An European Guideline and training standards for diabetes prevention (IMAGE). Diab. Vasc. Dis. Res..

[29] Bayındır Çevik A., Metin Karaaslan M., Koçan S., Pekmezci H., Baydur Şahin S., Kırbaş A., Ayaz T. (2016). Prevalence and screening for risk factors of type 2 diabetes in Rize, Nourtheast Turkey: Findings from a population-based study. Prim. Care Diabetes.

[30] Ephraim R.K.D., Owusu V.B., Asiamah J., Mills A., Abaka-Yawson A., Kpene G.E., Kwadzokpui P.K., Adusei S. (2020). Predicting type 2 diabetes mellitus among fishermen in Cape Coast: A comparison between the FINDRISC score and the metabolic syndrome. J. Diabetes Metab. Disord..

[31] Meijnikman A.S., De Block C.E.M., Verrijken A., Mertens I., Van Gaal L.F. (2018). Predicting type 2 diabetes mellitus: A comparison between the FINDRISC score and the metabolic syndrome. Diabetol. Metab. Syndr..

[32] International Diabetes Federation (2022). IDF Diabetes Atlas.

[33] American Diabetes Association (2024). Prevention or delay o diabetes and associated comorbidities: Standars of care in diabetes 2024. Diabetes Care.

[34] 2018 Physical Activity Guidelines Advisory Committee (2018). 2018 Physical Activity Guidelines Advisory Committee Scientific Report.

[35] Afshin A., Forouzanfar M.H., Reitsma M.B., Sur P., Estep K., Lee A. (2017). Health Effects of Overweight and Obesity in 195 Countries over 25 Years. N. Engl. J. Med..

[36] Sonmez A., Yumuk V., Haymana C., Demirci I., Barcin C., Kıyıcı S., Güldiken S., Örük G., Saydam B.O., Baldane S. (2019). Impact of Obesity on the Metabolic Control of Type 2 Diabetes: Results of the Turkish Nationwide Survey of Glycemic and Other Metabolic Parameters of Patients with Diabetes Mellitus (TEMD Obesity Study). Obes. Facts.

[37] Kriska A. (2000). Physical activity and the prevention of type 2 diabetes mellitus. Sports Med..

[38] Orozco L.J., Buchleitner A.M., Gimenez-Perez G., i Figuls M.R., Richter B., Mauricio D. (2008). Exercise or exercise and diet for preventing type 2 diabetes mellitus. Cochrane Database Syst. Rev..

[39] Feito Y., Patel P., Sal Redondo A., Heinrich K.M. (2019). Effects of eight weeks of high intensity functional training on glucose control and body composition among overweight and obese adults. Sports.

[40] Zhang Y., Pan X.-F., Chen J., Xia L., Cao A., Zhang Y., Wang J., Li H., Yang K., Guo K. (2020). Combined lifestyle factors and risk of incident type 2 diabetes and prognosis among individuals with type 2 diabetes: A systematic review and meta-analysis of prospective cohort studies. Diabetologia.

[41] Centers for Disease Control and Prevention National Diabetes Statistics Report, 2020: Estimates of Diabetes and Its Burden in the United States. https://www.cdc.gov/diabetes/pdfs/data/statistics/national-diabetes-statistics-report.pdf.

[42] Kulak E., Berber B., Temel H., Kutluay S.N., Yıldırım M., Dedeoğlu F.N., Cifcili S. (2019). Determining the risk level of type 2 diabetes in individuals applying to family medicine. Türk. Aile. Hek. Derg..

[43] Qian F., Liu G., Hu F.B., Bhupathiraju S.N., Sun Q. (2019). Association between plant-based dietary patterns and risk of type 2 diabetes: A systematic review and meta-analysis. JAMA Intern. Med..

[44] Ley S.H., Hamdy O., Mohan V., Hu F.B. (2014). Prevention and management of type 2 diabetes: Dietary components and nutritional strategies. Lancet.

[45] Jacobs S., Harmon B.E., Boushey C.J., Morimoto Y., Wilkens L.R., Le Marchand L., Kröger J., Schulze M.B., Kolonel L.N., Maskarinec G. (2015). A priori-defined diet quality indexes and risk of type 2 diabetes: The Multiethenic Cohort. Diabetología.

[46] Chiuve S.E., Fung T.T., Rimm E.B., Hu F.B., McCullough M.L., Wang M., Willett W.C. (2012). Alternative dietary indices both strongly predict risk of chronic disease. J. Nutr..

[47] Evert A.B., Dennison M., Gardner C.D., Garvey W.T., Lau K.H.K., MacLeod J., Mitri J., Pereira R.F., Rawlings K., Robinson S. (2019). Nutrition therapy for adults with diabetes or prediabetes: A consensus report. Diabetes Care.

