# Supplementary material for: Risk of Type 2 Diabetes in University Students at the University of Extremadura: A Cross-Sectional Study
Source: J Pers Med. 2024 Jan 29;14(2):146. doi: 10.3390/jpm14020146 (PMC10890267; doi:10.3390/jpm14020146)
Supplement: Supplementary file 1 [file jpm-14-00146-s001.zip › jpm-2816343-supplementary.pdf]

Correlations

			Score	Metabolic rate	% Fat mass	kg fat mass	% Fat-free mass	kg fat-free mass	% Lean mass	Kg lean mass	% Body water	Kg body water	% Bone minera	Kg bone mineral	Age	Height	Kcal basal metabolic	Weight	BMI	Sarcopenia risk index	Visceral fat index	Waist circumference	Hip perimeter	Waist hip index
Rho de Spearman	Score	Correlation coefficient	1,000	-,338**	,450**	,332**	-,445**	-0,024	-,439**	0,010	-,415**	-0,007	-,449**	0,007	,257**	-0,106	-0,005	,184**	,353**	0,046	,386**	,431**	,455**	,259*
		correlation Sig. (bilateral)		0,000	0,000	0,000	0,000	0,663	0,000	0,860	0,000	0,893	0,000	0,900	0,000	0,065	0,931	0,001	0,000	0,422	0,000	0,000	0,000	0,007
		N	341	286	302	338	302	337	301	302	300	337	301	290	221	302	306	306	303	300	287	107	107	107
	Metabolic rate	Correlation coefficient	-,338**	1,000	-,681**	-,840**	,684**	-,313**	,677**	-,302**	,600**	-,358**	,709**	-,302**	-0,135	-,145*	-,323**	-,604**	-,696**	-,289**	-,707**	-,563**	-,700**	-,239*
		correlation Sig. (bilateral)	0,000		0,000	0,000	0,000	0,000	0,000	0,000	0,000	0,000	0,000	0,000	0,055	0,015	0,000	0,000	0,000	0,000	0,000	0,000	0,000	0,013
		N	286	286	286	284	286	284	282	286	286	283	282	286	201	282	286	286	286	286	286	107	107	107
	% Fat Mass	Correlation coefficient	,450**	-,681**	1,000	,851**	-,992**	-,250**	-,995**	-,271**	-,886**	-,221**	-,965**	-,252**	0,095	-,373**	-,227**	,130*	,475**	-,236**	,417**	,431**	,562**	0,131
		correlation Sig. (bilateral)	0,000	0,000		0,000	0,000	0,000	0,000	0,000	0,000	0,000	0,000	0,000	0,163	0,000	0,000	0,024	0,000	0,000	0,000	0,000	0,000	0,180
		N	302	286	302	300	302	300	298	302	300	299	298	289	217	298	302	302	301	300	287	107	107	107
	Kg fat mass	Correlation coefficient	,332**	-,840**	,851**	1,000	-,847**	,449**	-,793**	,206**	-,831**	,471**	-,829**	,193**	,193**	0,011	,193**	,561**	,755**	,181**	,697**	,649**	,755**	,298*
		correlation Sig. (bilateral)	0,000	0,000	0,000		0,000	0,000	0,000	0,000	0,000	0,000	0,000	0,001	0,004	0,850	0,001	0,000	0,000	0,002	0,000	0,000	0,000	0,002
		N	338	284	300	338	300	337	301	300	298	337	301	288	219	302	304	304	301	298	285	107	107	107
	% Fat- free mass	Correlation coefficient	-,445**	,684**	-,992**	-,847**	1,000	,241**	,995**	,265**	,889**	,218**	,964**	,244**	-0,079	,371**	,221**	-,136*	-,478**	,235**	-,414**	-,415**	-,555**	-0,114
		correlation Sig. (bilateral)	0,000	0,000	0,000	0,000		0,000	0,000	0,000	0,000	0,000	0,000	0,000	0,249	0,000	0,000	0,018	0,000	0,000	0,000	0,000	0,000	0,244
		N	302	286	302	300	302	300	298	302	300	299	298	289	217	298	302	302	301	300	287	107	107	107
	kg fat-free mass	Correlation coefficient	-0,024	-,313**	-,250**	,449**	,241**	1,000	,277**	,977**	0,078	,973**	,170**	,952**	,207**	,793**	,888**	,859**	,528**	,820**	,476**	,716**	,603**	,506*
		correlation Sig. (bilateral)	0,663	0,000	0,000	0,000	0,000		0,000	0,000	0,180	0,000	0,003	0,000	0,002	0,000	0,000	0,000	0,000	0,000	0,000	0,000	0,000	0,000
		N	337	284	300	337	300	337	300	300	298	336	300	288	218	301	303	303	301	298	285	107	107	107
	% Lean mass	Correlation coefficient	-,439**	,677**	-,995**	-,793**	,995**	,277**	1,000	,265**	,895**	,258**	,967**	,231**	-0,104	,353**	,198**	-,139*	-,483**	,231**	-,418**	-,428**	-,559**	-0,128
		correlation Sig. (bilateral)	0,000	0,000	0,000	0,000	0,000	0,000		0,000	0,000	0,000	0,000	0,000	0,129	0,000	0,001	0,016	0,000	0,000	0,000	0,000	0,000	0,190
		N	301	282	298	301	298	300	301	298	296	301	301	286	216	300	301	301	298	296	283	107	107	107
	Kg lean mass	Correlation coefficient	0,010	-,302**	-,271**	,206**	,265**	,977**	,265**	1,000	0,105	,950**	,155**	,995**	,225**	,809**	,939**	,891**	,557**	,850**	,481**	,718**	,603**	,510*
		correlation Sig. (bilateral)	0,860	0,000	0,000	0,000	0,000	0,000	0,000		0,068	0,000	0,007	0,000	0,001	0,000	0,000	0,000	0,000	0,000	0,000	0,000	0,000	0,000
		N	302	286	302	300	302	300	298	302	300	299	298	289	217	298	302	302	301	300	287	107	107	107
	% Body water	Correlation coefficient	-,415**	,600**	-,886**	-,831**	,889**	0,078	,895**	0,105	1,000	,144*	,888**	0,094	-,165*	,184**	0,087	-,249**	-,498**	,186**	-,367**	-,465**	-,587**	-0,164
		correlation Sig. (bilateral)	0,000	0,000	0,000	0,000	0,000	0,180	0,000	0,068		0,013	0,000	0,111	0,016	0,001	0,134	0,000	0,000	0,001	0,000	0,000	0,000	0,091
		N	300	286	300	298	300	298	296	300	300	297	296	289	215	296	300	300	300	300	287	107	107	107
	kg body water	Correlation coefficient	-0,007	-,358**	-,221**	,471**	,218**	,973**	,258**	,950**	,144*	1,000	,155**	,934**	,166*	,712**	,848**	,845**	,562**	,877**	,525**	,699**	,589**	,496*
		correlation Sig. (bilateral)	0,893	0,000	0,000	0,000	0,000	0,000	0,000	0,000	0,013		0,007	0,000	0,014	0,000	0,000	0,000	0,000	0,000	0,000	0,000	0,000	0,000
		N	337	283	299	337	299	336	301	299	297	337	301	287	218	301	303	303	300	297	284	107	107	107
	% Bone mineral	Correlation coefficient	-,449**	,709**	-,965**	-,829**	,964**	,170**	,967**	,155**	,888**	,155**	1,000	,136*	-,135*	,259**	0,101	-,241**	-,556**	,139*	-,486**	-,486**	-,612**	-0,169
		correlation Sig. (bilateral)	0,000	0,000	0,000	0,000	0,000	0,003	0,000	0,007	0,000	0,007		0,021	0,047	0,000	0,080	0,000	0,000	0,017	0,000	0,000	0,000	0,083
		N	301	282	298	301	298	300	301	298	296	301	301	286	216	300	301	301	298	296	283	107	107	107
	Kg bone mineral	Correlation coefficient	0,007	-,302**	-,252**	,193**	,244**	,952**	,231**	,995**	0,094	,934**	,136*	1,000	,228**	,801**	,937**	,888**	,561**	,847**	,479**	,703**	,594**	,497*
		correlation Sig. (bilateral)	0,900	0,000	0,000	0,001	0,000	0,000	0,000	0,000	0,111	0,000	0,021		0,001	0,000	0,000	0,000	0,000	0,000	0,000	0,000	0,000	0,000
		N	290	286	289	288	289	288	286	289	289	287	286	290	205	286	290	290	290	289	287	107	107	107
	Age	Correlation coefficient	,257**	-0,135	0,095	,193**	-0,079	,207**	-0,104	,225**	-,165*	,166*	-,135*	,228**	1,000	0,078	0,122	,262**	,265**	,206**	,406**	,222*	,226*	0,164
		correlation Sig. (bilateral)	0,000	0,055	0,163	0,004	0,249	0,002	0,129	0,001	0,016	0,014	0,047	0,001		0,253	0,070	0,000	0,000	0,002	0,000	0,022	0,020	0,091
		N																						

	N	221	201	217	219	217	218	216	217	215	218	216	205	221	217	221	221	218	215	202	107	107	107
Height	Correlation coefficient	-0,106	-,145*	-,373**	0,011	,371**	,793**	,353**	,809**	,184**	,712**	,259**	,801**	0,078	1,000	,791**	,655**	,136*	,469**	,191**	,468**	,400**	,344**
	correlation Sig. (bilateral)	0,065	0,015	0,000	0,850	0,000	0,000	0,000	0,000	0,001	0,000	0,000	0,000	0,253		0,000	0,000	0,018	0,000	0,001	0,000	0,000	0,000
	N	302	282	298	302	298	301	300	298	296	301	300	286	217	302	302	302	299	296	283	107	107	107
Kcal basal metabolic	Correlation coefficient	-0,005	-,323**	-,227**	,193**	,221**	,888**	,198**	,939**	0,087	,848**	0,101	,937**	0,122	,791**	1,000	,844**	,515**	,783**	,435**	,737**	,624**	,517**
	correlation Sig. (bilateral)	0,931	0,000	0,000	0,001	0,000	0,000	0,001	0,000	0,134	0,000	0,080	0,000	0,070	0,000		0,000	0,000	0,000	0,000	0,000	0,000	0,000
	N	306	286	302	304	302	303	301	302	300	303	301	290	221	302	306	306	303	300	287	107	107	107
Weight	Correlation coefficient	,184**	-,604**	,130*	,561**	-,136*	,859**	-,139*	,891**	-,249**	,845**	-,241**	,888**	,262**	,655**	,844**	1,000	,786**	,773**	,701**	,835**	,783**	,532**
	correlation Sig. (bilateral)	0,001	0,000	0,024	0,000	0,018	0,000	0,016	0,000	0,000	0,000	0,000	0,000	0,000	0,000	0,000		0,000	0,000	0,000	0,000	0,000	0,000
	N	306	286	302	304	302	303	301	302	300	303	301	290	221	302	306	306	303	300	287	107	107	107
BMI	Correlation coefficient	,353**	-,696**	,475**	,755**	-,478**	,528**	-,483**	,557**	-,498**	,562**	-,556**	,561**	,265**	,136*	,515**	,786**	1,000	,658**	,797**	,768**	,746**	,460**
	correlation Sig. (bilateral)	0,000	0,000	0,000	0,000	0,000	0,000	0,000	0,000	0,000	0,000	0,000	0,000	0,000	0,018	0,000	0,000		0,000	0,000	0,000	0,000	0,000
	N	303	286	301	301	301	301	298	301	300	300	298	290	218	299	303	303	303	300	287	107	107	107
Sarcopenia risk index	Correlation coefficient	0,046	-,289**	-,236**	,181**	,235**	,820**	,231**	,850**	,186**	,877**	,139*	,847**	,206**	,469**	,783**	,773**	,658**	1,000	,555**	,621**	,455**	,528**
	correlation Sig. (bilateral)	0,422	0,000	0,000	0,002	0,000	0,000	0,000	0,000	0,001	0,000	0,017	0,000	0,002	0,000	0,000	0,000	0,000		0,000	0,000	0,000	0,000
	N	300	286	300	298	300	298	296	300	300	297	296	289	215	296	300	300	300	300	287	107	107	107
Visceral fat index	Correlation coefficient	,386**	-,707**	,417**	,697**	-,414**	,476**	-,418**	,481**	-,367**	,525**	-,486**	,479**	,406**	,191**	,435**	,701**	,797**	,555**	1,000	,645**	,686**	,389**
	correlation Sig. (bilateral)	0,000	0,000	0,000	0,000	0,000	0,000	0,000	0,000	0,000	0,000	0,000	0,000	0,000	0,001	0,000	0,000	0,000	0,000		0,000	0,000	0,000
	N	287	286	287	285	287	285	283	287	287	284	283	287	202	283	287	287	287	287	287	107	107	107
Waist circunference	Correlation coefficient	,431**	-,563**	,431**	,649**	-,415**	,716**	-,428**	,718**	-,465**	,699**	-,486**	,703**	,222*	,468**	,737**	,835**	,768**	,621**	,645**	1,000	,695**	,786**
	correlation Sig. (bilateral)	0,000	0,000	0,000	0,000	0,000	0,000	0,000	0,000	0,000	0,000	0,000	0,000	0,022	0,000	0,000	0,000	0,000	0,000	0,000		0,000	0,000
	N	107	107	107	107	107	107	107	107	107	107	107	107	107	107	107	107	107	107	107	107	107	107
Hip circunference	Correlation coefficient	,455**	-,700**	,562**	,755**	-,555**	,603**	-,559**	,603**	-,587**	,589**	-,612**	,594**	,226*	,400**	,624**	,783**	,746**	,455**	,686**	,695**	1,000	0,169
	correlation Sig. (bilateral)	0,000	0,000	0,000	0,000	0,000	0,000	0,000	0,000	0,000	0,000	0,000	0,000	0,020	0,000	0,000	0,000	0,000	0,000	0,000	0,000		0,082
	N	107	107	107	107	107	107	107	107	107	107	107	107	107	107	107	107	107	107	107	107	107	107
Waist to hip ratio	Correlation coefficient	,259**	-,239*	0,131	,298**	-0,114	,506**	-0,128	,510**	-0,164	,496**	-0,169	,497**	0,164	,344**	,517**	,532**	,460**	,528**	,389**	,786**	0,169	1,000
	correlation Sig. (bilateral)	0,007	0,013	0,180	0,002	0,244	0,000	0,190	0,000	0,091	0,000	0,083	0,000	0,091	0,000	0,000	0,000	0,000	0,000	0,000	0,000	0,082	
	N	107	107	107	107	107	107	107	107	107	107	107	107	107	107	107	107	107	107	107	107	107	107

**. Correlation is significant at the 0.01 level (bilateral).
*. Correlation is significant at the 0.05 level (bilateral).
